# Genome-wide identification, characterization, interaction network and expression profile of *GRAS* gene family in sweet orange (*Citrus sinensis*)

**DOI:** 10.1038/s41598-018-38185-z

**Published:** 2019-02-15

**Authors:** Hua Zhang, Limin Mi, Long Xu, Changxiu Yu, Chen Li, Chunli Chen

**Affiliations:** 1 College of Life Science and Technology, Huazhong Agricultural University, Wuhan, 430070 China; 2 School of Basic Medicine, Hubei University of Medicine, Shiyan, Hubei 442000 China; 3 Key Laboratory of Horticultural Plant Biology (Ministry of Education), Huazhong Agricultural University, Wuhan, 430070 China; 4 College of Resources and Environment, Huazhong Agricultural University, Wuhan, 430070 China

## Abstract

*GRAS* genes are suggested to be grouped into plant-specific transcriptional regulatory families that have been reported to participate in multiple processes, including plant development, phytohormone signaling, the formation of symbiotic relationships, and response to environmental signals. *GRAS* genes have been characterized in a number of plant species, but little is known about this gene family in *Citrus sinensis*. In this study, we identified a total of 50 *GRAS* genes and characterized the gene structures, conserved motifs, genome localizations and cis-elements within their promoter regions. According to their structural and phylogenetic features, the identified sweet orange *GRAS* members were divided into 11 subgroups, of which subfamily *CsGRAS34* was sweet orange-specific. Based on publicly available RNA-seq data generated from callus, flower, leaf and fruit in sweet orange, we found that some sweet orange *GRAS* genes exhibited tissue-specific expression patterning. Three of the six members of subfamily AtSHR, particularly *CsGRAS9*, and two of the six members of subfamily AtPAT1 were preferentially expressed in leaf. Moreover, protein-protein interactions with CsGRAS were predicted. Gene expression analysis was performed under conditions of phosphate deficiency, and GA3 and NaCl treatment to identify the potential functions of *GRAS* members in regulating stress and hormone responses. This study provides the first comprehensive understanding of the *GRAS* gene family in the sweet orange genome. As such, the study generates valuable information for further gene function analysis and identifying candidate genes to improve abiotic stress tolerance in citrus plants.

## Introduction

*C. sinensis* is an extremely important fruit crop in many countries. The release of the whole-genome sequence of sweet orange provides an opportunity to comprehensively analyze numerous known gene families^1,2^. GRAS proteins, which are plant-specific transcription factors, contain a variable N-terminal domain and five distinct highly conserved motifs in the C-terminus: LRІ, VHIID, LRII, PFYRE and SAW^3,4^. The intrinsically disordered N-terminal regions are likely responsible for the specific function of each *GRAS* gene^5^. GRAS proteins, were classified into ten subfamilies based on their structural characteristics: DELLA, AtLAS, AtSCR, AtSHR, AtPAT1, HAM, LISCL, AtSCL3, SCL4/7 and DLT^5^, and were found to play crucial roles in diverse fundamental processes of plant growth and development^5^.

The most widely known biological function of the GRAS family is establishing radial root patterning. For instance, AtSCR (scarecrow), mainly expressed in the cortex/endodermal initial cells, is involved in radial root patterning and the distal specification of the quiescent center (QC)^6,7^. Analysis of the short-root (*shr*) mutant showed that the AtSHR protein is also required for asymmetric cell division, responsible for formation of ground tissue (endodermis and cortex) as well as specification of endodermis in Arabidopsis root^8^. AtSCARECROW-LIKE23 (AtSCL23), a mobile protein, controls movement of SHR and acts redundantly with SCR to specify endodermal fate in the root meristem^9^. GRAS proteins function in axillary meristem initiation, shoot meristem maintenance and male gametogenesis. Tomato Lateral suppressor (Ls), Arabidopsis LATERAL SUPPRESSOR (LAS) and rice monoclum 1 (MOC1) are orthologous proteins regulating axillary meristem initiation and outgrowth^10–12^. TaMOC1, a putative MOC1 ortholog in wheat, is associated with wheat spikelet development^13^. In the petunia mutant hairy meristem (ham), shoot apical meristems fail to retain their undifferentiated character^14^. LlSCL (Liliumlongiflorum Scarecrow-like) is involved in transcriptional regulation during microsporogenesis within the lily anther^15^.

The GRAS gene family also participates in phytohormone signaling pathways such as gibberellin acid (GA), jasmonic acid (JA) and brassinosteroid (BR) signal transduction. For example, AtGAI (GA-insensitive), AtRGA (repressor of GA), AtRGL1 (RGA-like 1) and AtRGL2 (RGA-like 2), well-known members of the DELLA subfamily of GRAS proteins, function as negative regulators of GA responses^16–19^. Overexpression of OsGAI in rice and tobacco modulates GA-dependent multiple responses such as increasing the number of tillers in rice^20^. AtSCL3 (scarecrow like 3) is a positive regulator of the GA response pathway^21^. AtRGL3 (RGA-like 3) regulates jasmonic acid (JA) signaling^22^. DLT (dwarf and low-tillering) and OsGRAS19 act as positive regulators in brassinosteroid signaling in rice^23,24^. In addition, GRAS proteins are involved in the formation of symbiotic relationships. OsSLR1, the AtGAI homolog in rice, not only acst as an intermediate of the GA-signal transduction pathway but also participates in arbuscular mycorrhizal symbiosis in plants by forming a DELLA complex with DIP1 (DELLA Interacting Protein 1). Nodulation Signaling Pathway1 (NSP1) and NSP2 are involved in early Nod-factor signaling by forming a complex in the model legume *Medicago truncatula*. Within this complex, NSP1 binds directly to early nodulin gene ENOD11 promoters through the novel cis-element AATTT^25–27^. The GRAS-type transcriptional regulators NSP1 and NSP2 were also required in carotenoid isomerase gene DWARF27 co-opted in rhizobium symbiosis and strigolactone (SL) biosynthesis in *M. truncatula* and rice^28,29^. Phosphorus (Pi), is an important macronutrient for all plants, including citrus. Low phosphate (Pi) availability exists in both natural and agricultural ecosystems. In response to Pi deficiency, plants modify their root architecture to improve Pi acquisition by reducing growth of primary roots and increasing the number and length of lateral roots (LRs) and root hairs^30–32^. Pi deficiency negatively impactes growth and development of citrus. Pi deficient plants exhibite reduced flowering, bronzed and smaller leaves, smaller fruit with reduced juice, and weak branches. *Poncirus trifoliata* (L.) Raf is relative to citrus and widely used as a root stock of citrus^33^. A majority of genes is involved in response to Pi deficiency^34^.

GRAS proteins also function in responding to environmental signals such as light and abiotic/biotic stress. SCL21 (SCARECROW-LIKE21) and PAT1 (PHYTOCHROME A SIGNAL TRANSDUCTION1) are positive regulators of phytochrome A (phyA) signal transduction for several high-irradiance responses^35,36^. AtSCL13 is a positive regulator of continuous red light signaling downstream of phytochrome B (phyB)^37^. SCL14 serves as a transcriptional coactivator of TGA transcription factors and regulates the induction of genes involved in the detoxification of harmful chemicals^38^. OsCIGR1 and OsCIGR2 act as transcriptional regulators in the early events of the elicitor-induced defense response in rice^39^. OsGRAS23, a rice GRAS transcription factor, positively regulates rice drought tolerance via the induction of a number of stress-responsive genes^40^. *VaPAT1*, one GRAS gene of *Vitis amurensis*, is induced by cold, drought and high salinity but repressed by exogenous gibberellic acid. *VaPAT1* overexpression in Arabidopsis increases tolerance to cold, drought and high salinity, with higher levels of proline and soluble sugar under stress treatment in seedlings^41^. *PeSCL7*, a poplar GRAS/SCL gene, is also induced by drought and high salt, and repressed by gibberellic acid (GA) treatment. Compared with wide-type plant, Arabidopsis overexpressing *PeSCL7* shows higher tolerance to drought and salt treatment^2^.

To date, the *GRAS* gene family has been identified and characterized in rice^42^, Arabidopsis^43^, Chinese cabbage^44^, Populus^45^, pine^46^, Prunusmume^47^, tomato^48^, tobacco^49^, grapevine^50^ and tea plant^51^. However, there is little information on the identification and functional characterization of GRAS proteins in citrus, the most important evergreen perennial fruit tree. Here we present the first detailed and comprehensive analyses of *GRAS* gene family in the whole genome of sweet orange. The present work identified 50 putative *CsGRAS* genes in *C. sinensis*, together with analyzing their gene characters and chromosome distribution. Then, we discerned phylogenetic relationships between *C. sinensis*, Arabidopsis and rice, noting main biological functions of GRAS proteins from recent studies. Subsequently, we performed RNA-Seq and qRT-PCR analysis to confirm the tissue expression patterns. Gene-gene interations with *CsGRAS* were also analyzed. Next, their transcript abundance in response to Pi deficiency treatments in *P. Trifoliata* (L.) Raf was investigated by both RNA-Seq and qRT-PCR analyses. In addition, the transcriptional level of *CsGRAS* genes in response to GA_3_ and NaCl was examined. This study provides details of the *GRAS* gene family and facilitates the further functional characterization of GRAS genes in Citrus.

## Result and Discussion

### Structural Analysis of CsGRAS Family

In order to run a complete search for identifying *GRAS* genes in the genome of sweet orange, all annotated proteins of the genome from sweet orange annotation project database of Huazhong Agricultural University (http://citrus.hzau.edu.cn/orange/) and the phytozome *C. sinensis* (v1.1) database (http://phytozome.jgi.doe.gov/-/pz/portal.html) were considered analyzed. The Hidden Markov Model (HMM) profile of the GRAS domain (PF03514) (http://pfam.sanger.ac.uk/) was then employed as a query to search the database using the program HMM3.0 with the default E-value. After determining the integrity of the GRAS domain of GRAS proteins by using the online program SMART (http://smart.embl-heidelberg.de/) and sequence alignment, 50 genes (Table S1) were assigned as *GRAS* genes in *C. sinensis*, and named from *CsGRAS1* to *CsGRAS50* based on the coordinate order on *C. sinensis* chromosomes starting at the top of chromosome 1 from top to bottom.

**Figure 1:**
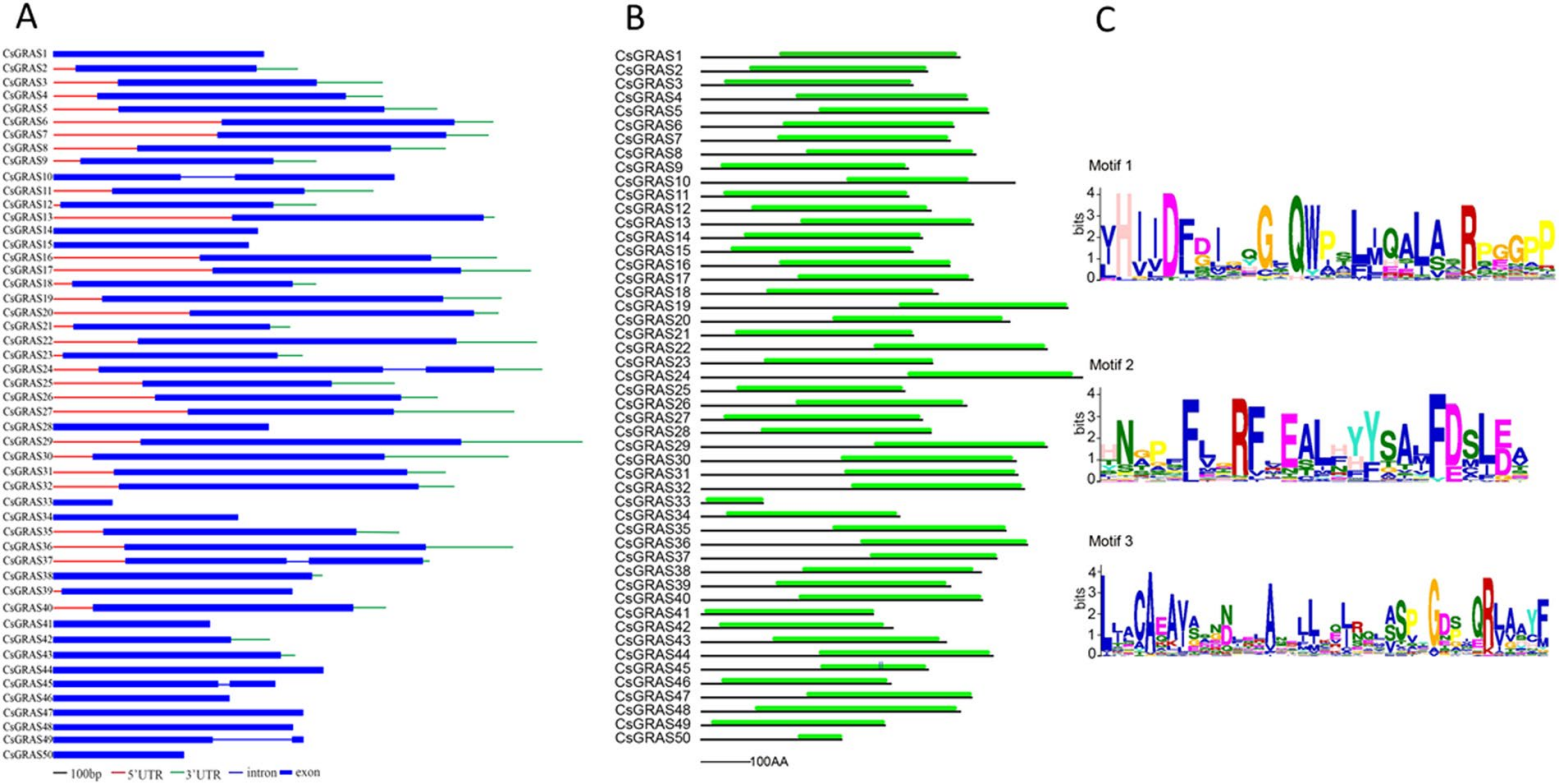
Structure of *CsGRAS* genes and CsGRAS proteins. (**A**) The gene structure based on the sequences of *GRAS* downloaded from orange genome database (http://citrus.hzau.edu.cn/orange/). (**B**) The protein structures based on the presence of GRAS domain as identified by SMART (http://smart.embl-heidelberg.de/). (**C**) Three conserved LOGOs for GRAS domain using the MEME algorithm (http://meme-suite.org/tools/meme).

For gene families, the patterns of exon/intron positions may play important roles in the process of evolution^52^. To examine the diversity of exon/intron patterns in *CsGRAS* genes, we conducted an exon/intron organization analysis in 50 *CsGRAS* genes (Fig. 1A). As in *Prunus mume* and Arabidopsis, many *CsGRAS* genes (45 genes) were intron-less, and 5 genes had one intron. Moreover, the results also showed that genes in the same branch may showed similar exon/intron organization. The number of *GRAS* genes in citrus (50 genes) was close to the number of *GRAS* genes in *P. mume* (46 *GRAS* genes), Chinese cabbage (48 *GRAS* genes), tomato (53 *GRAS* genes) and Oryza sativa (57 *GRAS* genes), which is more than that in Arabidopsis (33 *GRAS* genes), pine (32 *GRAS* genes). It was, however, less than that of *Populus* (106 *GRAS* genes), *Malus domestica* (127 *GRAS* genes), *Medicago truncatula* (75 *GRAS* genes), and *Musa acuminate* (73 *GRAS* genes) (Lu *et al*. 2015).In order to study the structure of CsGRAS proteins, SMART (http://smart.embl-heidelberg.de/) was employed to identify the GRAS domain in the 50 proteins. All of the CsGRAS proteins showed the GRAS domain, and the GRAS domains were located at the end of the CsGRAS proteins (Fig. 1B). In order to identify the conserved motifs between the 50 CsGRAS proteins, the MEME motif search tool was used to find the 3 conserved motifs in the GRAS domain (Fig. 1C). The 3 conserved motifs were located at the middle part, near the end part and the beginning, respectively. Collectively, GRAS domains had high similarity in the 50 CsGRAS proteins.

### Phylogenetic analysis of GRAS proteins in *C. sinensis* with Arabidopsis and rice

**Figure 2:**
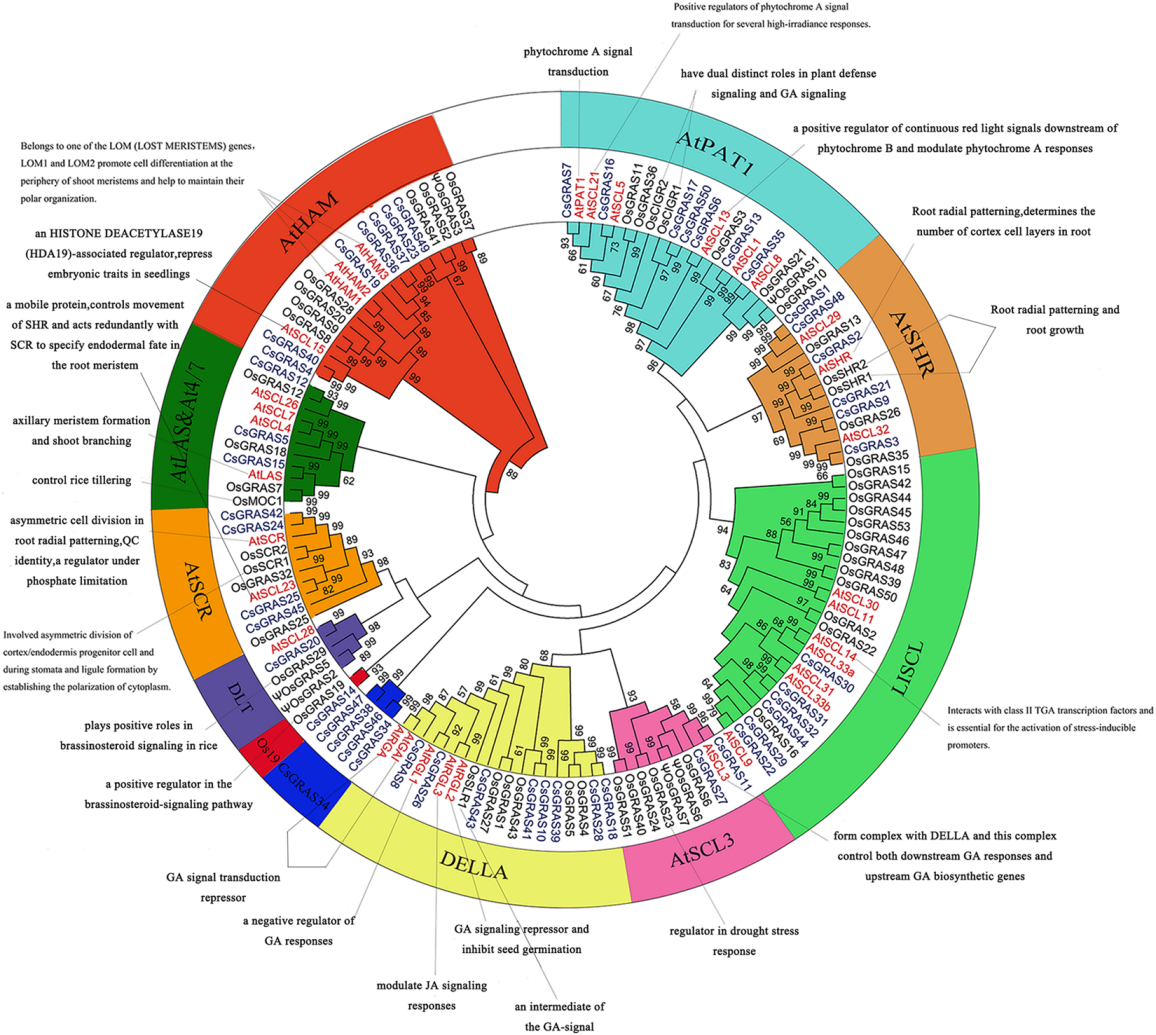
The phylogenetic tree of GRAS proteins among sweet orange, Arabidopsis and rice. Combined phylogenetic analysis of GRAS proteins from *C. sinensis* (Cs), *A. thaliana* (At) and *O. sativa* (Os). The GRAS proteins are clustered into 11 subgroups, marked by different colors. The main biological functions of some GRAS proteins, which are experimentally characterized along with some representative references, are shown in the phylogenetic tree.

To reconstruct the evolutionary history of the GRAS gene family in the studied plant species, we built a phylogenetic tree from the alignment of 149 full-length GRAS protein sequences in sweet orange (49 GRAS proteins), Arabidopsis (33 GRAS proteins) and rice (59 GRAS proteins) using NJ method (Fig. 2). In particular, CsGRAS33 was not included in phylogenetic tree analysis, because its protein sequence was too short with length of 136 amino acid (Table S1). This tree showed that CsGRAS proteins of the 3 species could be divided into 11 subfamilies. These subfamilies were designated according to earlier previous studies^5,42^ or named according to one of their members in the case of newly identified subfamilies. The eleven subfamilies were AtHAM, AtLAS & AtSCL4/7, AtSCR, DLT, Os19, CsGRAS34, DELLA, AtSCl3, LISCL, AtSHR, and AtPAT1. Remarkably, the CsGRAS34 subfamily, consisting of CsGRAS47/CsGRAS38/CsGRAS46/CsGRAS34, was distinguished from other GRAS genes since they form an individual clade, which suggested that this individual clade was specific to sweet orange, and there was no homolog member in rice. This meant that they were the result of duplication after the ancestors of Arabidopsis and citrus segregated. Os19 belonged to sweet orange and a rice-specific subfamily containing only two members (OsGRAS19 and CsGRAS14) that form a small unique monophyletic clade. In general, the majority of subfamilies harbored GRAS members from each of the three species (Fig. 2). However, no Arabidopsis gene existed in Os19 subfamily, indicating lineage-specific gene loss in Arabidopsis. Coincidently, a similar result occurred in combined phylogenetic analysis of GRAS protein in Populus^45^. Notably, the subfamily CsGRAS34 was sweet orange-specific, implying that it had been gained in the sweet orange lineage after divergence from the most recent common ancestor with Arabidopsis and rice or entirely lost from the latter two lineages. It was tempting to speculate that this subfamily had specialized roles in the adaptive evolution of sweet orange. Considering the conserved characteristics of structure and function, we summarized the main biological functions of GRAS proteins in plant development, using information from recent studies (Fig. 2).

Classification of orthologous GRAS will facilitate the future study of their functions in citrus, as evidenced by Ls/LAS/MOC1^10,11^, AtSHR/OsSHR1/OsSHR2/BdSHR^8,53,54^ and AtGAI/AtRGA/OsSLR1^16,17,55^. Thus, according to previous reports on AtSHR, AtSCR^6–8,53,54,56^, CsGRAS2, CsGRAS24 and CsGRAS42 are thought to be related to root patterning. CsGRAS7, the homolog of AtPAT1, may be connected with the phytochrome A-specific signaling pathway^35^. AtGAI and AtRGA function as a negative regulator of GA responses^16,17^, so it could be deduced that CsGRAS8 and CsGRAS26 are possibly involved in GA responses. CsGRAS15, the homology of Ls/LAS/MOC1, was associated with axillary meristem formation^10–12^. CsGRAS14, Os19 family member, possibly participates in brassinosteroid-signaling^24^. These results suggested the potential function of CsGRAS proteins which may have similar roles to other GRAS proteins of rice and Arabidopsis in the same subfamilies.

**Figure 3:**
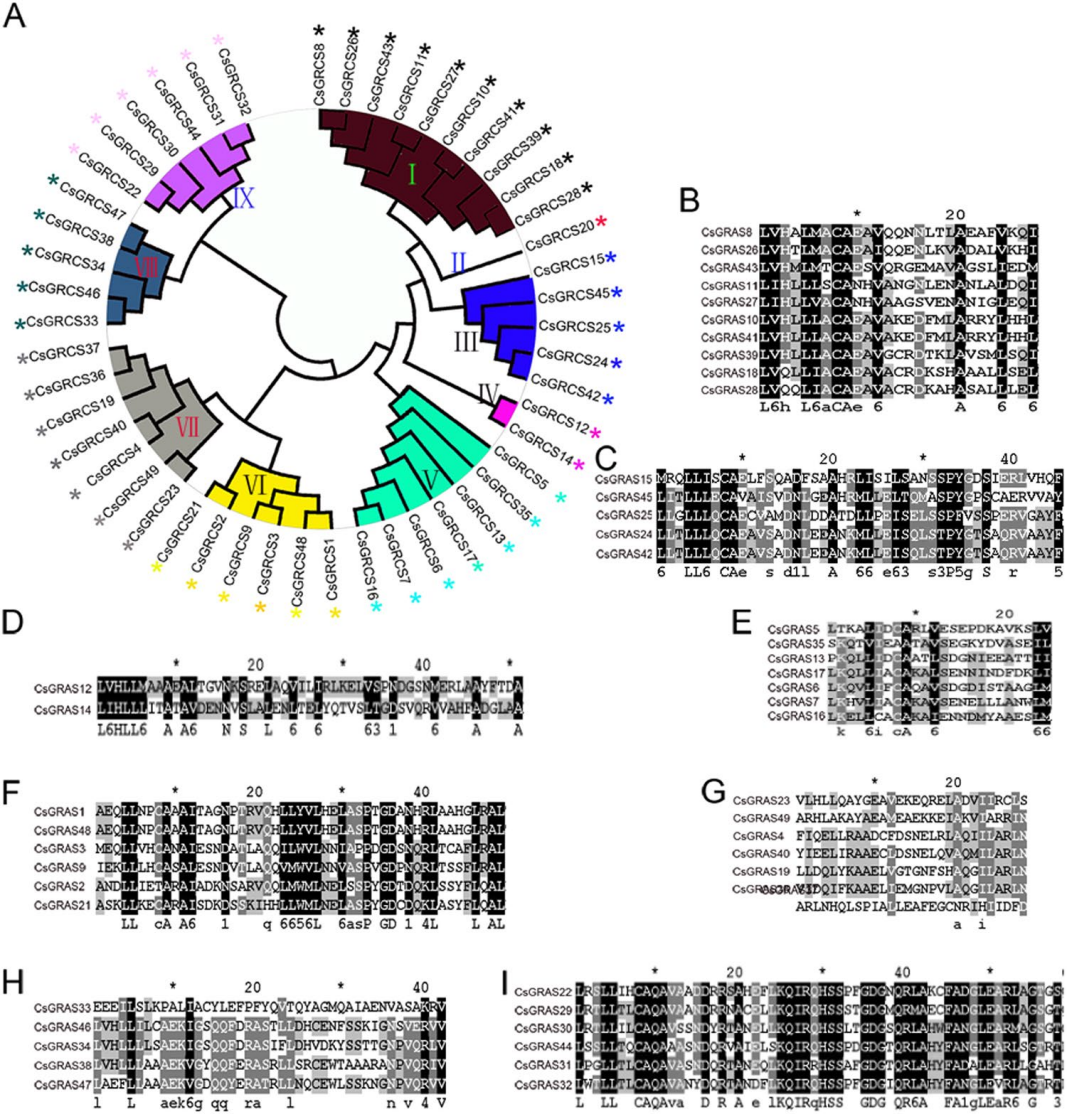
The phylogenetic analysis and Multiple sequence alignment of CsGRAS proteins. (**A**) Unrooted neighbour-joining phylogenetic tree of the CsGRAS family. The phylogenetic tree was generated using the MEGA 5.1 software (Tamura *et al*. 2011). (**B–I**) Conserved sequence of multiple sequence alignment of the CsGRAS proteins in subgroup I (**B**), subgroup III (**C**), subgroup IV (**D**), subgroup V (**E**), subgroup VI (**F**), subgroup VII (**G**), subgroup VIII (**H**), and subgroup IX (**I**) obtained using GneneDoc and ClustalX.

In order to study phylogenetic relationship between members of CsGRAS proteins, we also constructed a phylogenetic tree with GRAS domain sequences of 49 CsGRAS proteins. 9 subfamilies were generated, which were similar to a phylogenetic tree with full-length GRAS protein sequences of CsGRAS proteins, OsGRAS proteins and AtGRAS proteins (Fig. 3A). For example, CsGRAS proteins of subgroup IX were in LISCL family, and CsGRAS proteins of subgroup IV belonged to AtSHR family. To examine the conserved amino acids of GRAS domains in each subgroup, we constructed a multiple sequence alignment of GRAS domains of CsGRAS proteins in each subgroup. GRAS domain sequences in each subgroup showed high homology with each other. Moreover, the GRAS domain sequences of CsGRAS proteins in subgroup I (Fig. 3B), III (Fig. 3C), IV (Fig. 3D), V (Fig. 3E), VI (Fig. 3F), VIII (Fig. 3H) and IX (Fig. 3I) were significantly conserved within each subgroup, indicating that the proteins within each group may perform similar functions. In contrast, subgroup VII (Fig. 3G) proteins displayed reduced sequence similarity, suggesting these proteins had distinct origins and functions.

The goal of *GRAS* gene family genome-wide analysis in sweet orange was to make comprehensive predictions of citrus *GRAS* gene function. The location of a *CsGRAS* gene in the same branch of the phylogenetic tree as say the homologous gene in Arabidopsis or rice could give an approximate indication of the function of the *CsGRAS* gene in citrus. For example, the orthologous protein of AtPAT1, CsGRAS7, may therefore be involved in phyA signaling. The SCR-SHR complex controlled endodermis development in Arabidopsis^9^; however, citrus are woody plants with multiple cortex layers and are different to the single cortex in Arabidopsis. Therefore, the mechanism of SCR-SHR in citrus may be dissimilar to that in Arabidopsis. PtSHR2B in Populus functions in the regulation of phellem and periderm formation^57^; therefore, the homologous gene in citrus, CsGRAS2, may similarly function in phellem and periderm formation. The PPI network provided further information on the molecular function of the CsGRAS protein and confirmed predictions from the phylogenetic tree analysis. The protein which had protein-protein interaction with CsGRAS6 is calmodulin. Since CsGRAS6 belonged to AtPAT1 subfamily, it may also be involved in phyA or phyB signaling.

### Chromosomal distribution and gene duplication of *CsGRAS* genes

**Figure 4:**
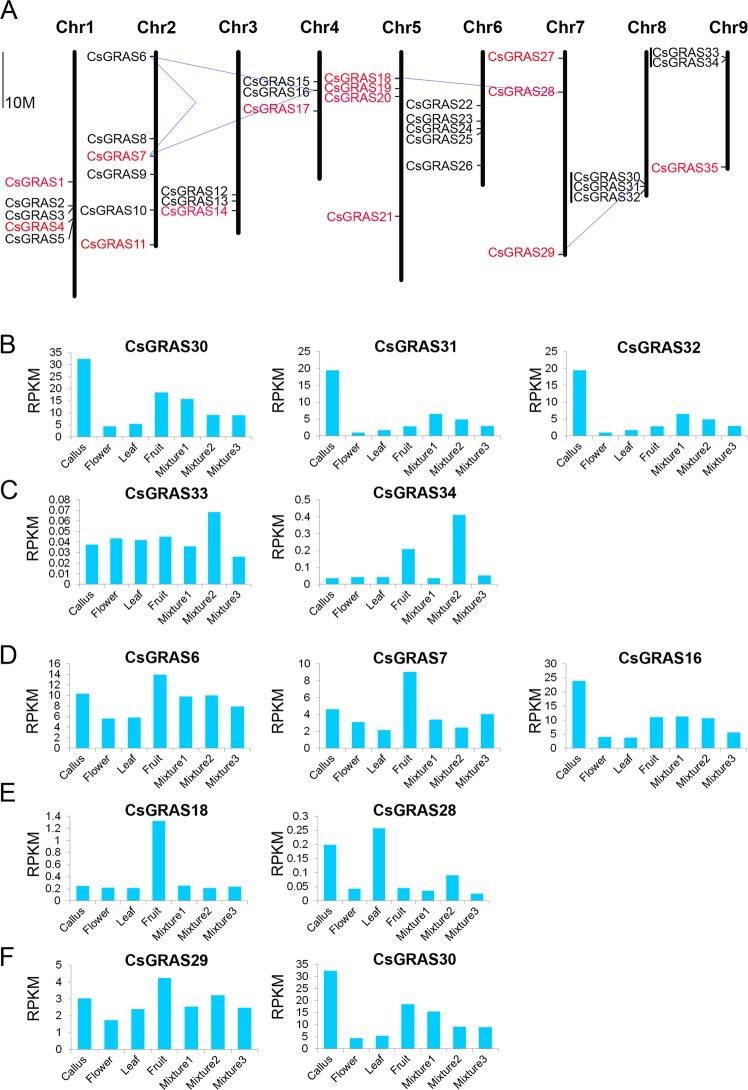
Genomic distribution of *CsGRAS* genes and expression patterns of CsGRAS duplicated genes. (**A**) The scale on the left is 10 megabases (10 Mb). Chromosome number is indicated at the top of each chromosome. Black and red show the forward and backward direction of transcription. The *CsGRAS* genes representing segmentally duplicated genes are connected by blue lines and the tandem duplicated genes are highlighted by dark lines on the left. The position of each *CsGRAS* gene on chromosome pseudomolecules in base pairs are given in Table S1 in electronic supplementary material. The expression levels of tandem duplicated *GRAS* genes (**B,C**) and segmentally duplicated *GRAS* genes (**D–F**) were analysed in *C. sinensis* annotation project database of Huazhong Agricultural University (http://citrus.hzau.edu.cn/orange/).

The physical positions of *GRAS* genes were obtained from the sweet orange annotation project database of Huazhong Agricultural University and the phytozome *Citrus sinensis* v1.1 *Citrus sinensis* database. The position and transcriptional direction of each gene are shown (Fig. 4A), and the exact positions on *C. sinensis* chromosome pseudomolecules are given in Table S1. The results show that 35 *CsGRAS* genes are distributed on 9 chromosomes, and that the tandem duplication genes, (*CsGRAS30*, *CsGRAS31* and *CsGRAS32*; *CsGRAS33* and *CsGRAS34*) are located on chromosome 8 and 9, as shown by connected dark lines in Fig. 4A. The segmental duplication genes are located on chromosome 2, 4, 5, 7 and 8, and indicated by connected blue lines (*CsGRAS6*, *CsGRAS7* and *CsGRAS16*; *CsGRAS18* and *CsGRAS28*; *CsGRAS29* and *CsGRAS30*) (Fig. 4A). The expression patterns of segmentally duplicated and tandemly duplicated *CsGRAS* genes were examined by using data from the *Citrus sinensis* annotation project database of Huazhong Agricultural University. The chromosome distribution of GRAS genes showed tandem repeats and segmental repeats, indicating that the derivation of *GRAS* genes in *C. sinensis* was generated by generic duplication and recombination.

It was found that one pair of tandem duplication genes and two pairs of segmental duplication genes had similar expression patterns. All of the three tandem duplication genes, *CsGRAS30*, *CsGRAS31* and *CsGRAS32*, had higher transcription levels in callus, but lower expression levels in other tissues (Fig. 4B). *CsGRAS33* was expressed in the 4 tissues with similar expression levels, whereas *CsGRAS34* showed higher transcriptional level in fruit (Fig. 4C). Moreover, *CsGRAS6*, *CsGRAS7* and *CsGRAS16* showed higher transcription accumulation in callus and fruit than in flower, leaf, and mixtures (Fig. 4D). Similarly, both of *CsGRAS29* and *CsGRAS30* also showed higher expression in callus and fruit (Fig. 4F). However, *CsGRAS18* and *CsGRAS28* displayed significantly different expression patterns (Fig. 4D). These results indicate that copies of duplicated genes with similar expression patterns might be functionally redundant, maintaining their functions during evolution, while the duplicated genes with different expression patterns may play distinct roles, suggesting that some members may have changed their function during the course of evolution^58^.

### Expression profiling of *CsGRAS* genes

**Figure 5:**
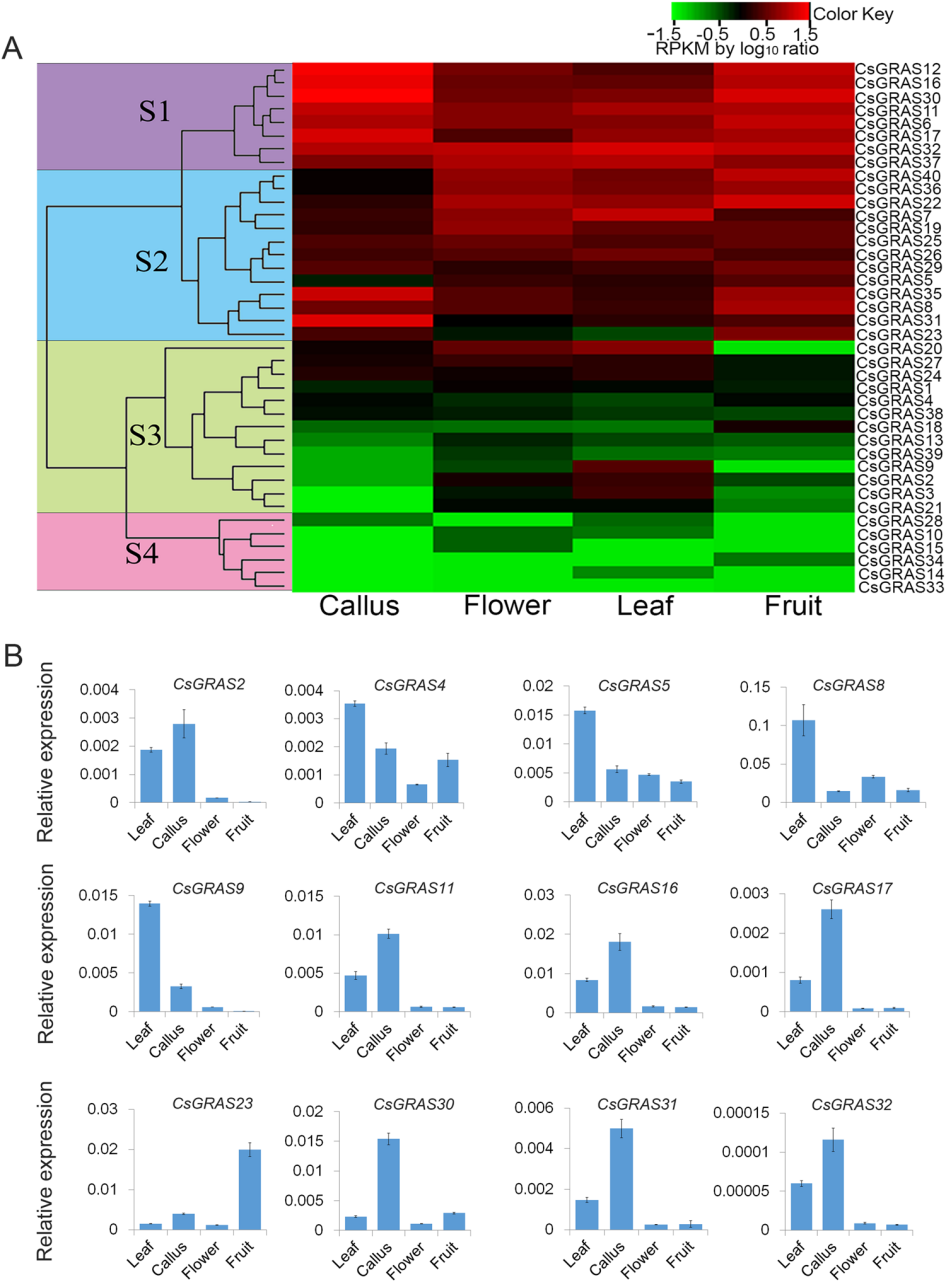
The expression pattern of 40 *GRAS* genes in sweet orange. (**A**) This heatmap was classified to 4 series. The data came from RNA-seq data (RPKM) and was transformed by log10 fold change. (**B**) Relative expression of 12 *CsGRAS* genes in 4 tissues of sweet orange, including callus, flower, leaf and fruit. Error bars denotes the standard deviation calculated from three independent experiments, with the Citrus actin gene as internal control.

To investigate the transcript accumulation of *CsGRAS* genes in the sweet orange, the expression profiling covering 4 tissues in *C. sinensis* were analyzed using Illumina GAII sequencer data from *Citrus sinensis* annotation project database of Huazhong Agricultural University^1^. The red or green colors represented the higher or lower relative abundance of each transcript in each sample, respectively, compared to the median expression value of that gene in the whole sample set. The expression patterns of *CsGRAS* genes could be classified into two major groups (Fig. 5A). Group 1, which contained subgroup S1 and S2, shows high transcript accumulations in the tissues analyzed. Some genes, such as *CsGRAS7*, *CsGRAS25*, *CsGRAS26* and *CsGRAS35*, had high expression level in four tissues. However, some genes in group 1 displayed high expression signals and preferential expression in some tissues, like *CsGRAS17* and *CsGRAS31*. *CsGRAS5* was abundantly expressed in leaf and callus, but showed low expression level in flower and fruit. *CsGRAS31* showed high transcriptional level in callus, but lower in leaf, flower and fruit. Group 2 contained 19 genes which could be divided into two subgroups, S3 and S4. Subgroup S4, which had low expression levels in nearly all tissues, especially in callus and fruit, consisted of 6 genes (C*sGRAS10, CsGRAS14, CsGRAS15, CsGRAS28, CsGRAS33* and *CsGRAS34*). *CsGRAS33* exhibited the lowest expression signals in all tissues. Subgroup S3 had 13 *CsGRAS* genes, all of which showed relatively low expression levels in most tissues analyzed, but higher in particular tissues, such as *CsGRAS2, CsGRAS3* and *CsGRAS9* in leaves, and *CsGRAS18* in fruit. *CsGRAS20, CsGRAS24* and *CsGRAS27* showed lower expression level in fruit, but higher in the 3 other tissues.

The expression patterns of 35 *CsGRAS* genes were further confirmed by real-time PCR analysis (Figs 5B and S1). The expression levels of *CsGRAS2, CsGRAS4, CsGRAS5, CsGRAS8*, *CsGRAS9* and *CsGRAS20* were enriched in leaf (Fig. 5B). Moreover, seven genes including *CsGRAS11, CsGRAS 16, CsGRAS 17, CsGRAS 30, CsGRAS 31* and *CsGRAS32* showed the highest expression level in callus (Fig. 5B), and *CsGRAS23* showed a high expression level in fruit (Fig. 5B). These results were consistent with RNA-seq data analysis. However, *CsGRAS1, CsGRAS24* and *CsGRAS 27* were highly expressed in callus (Fig. S1), which differed from the RNA-seq results. Taken together, the *GRAS* genes showed varied expression patterning in citrus tissues, implying multiple roles in citrus development.

### Interaction analysis between GRAS proteins in *C. sinensis*

**Figure 6:**
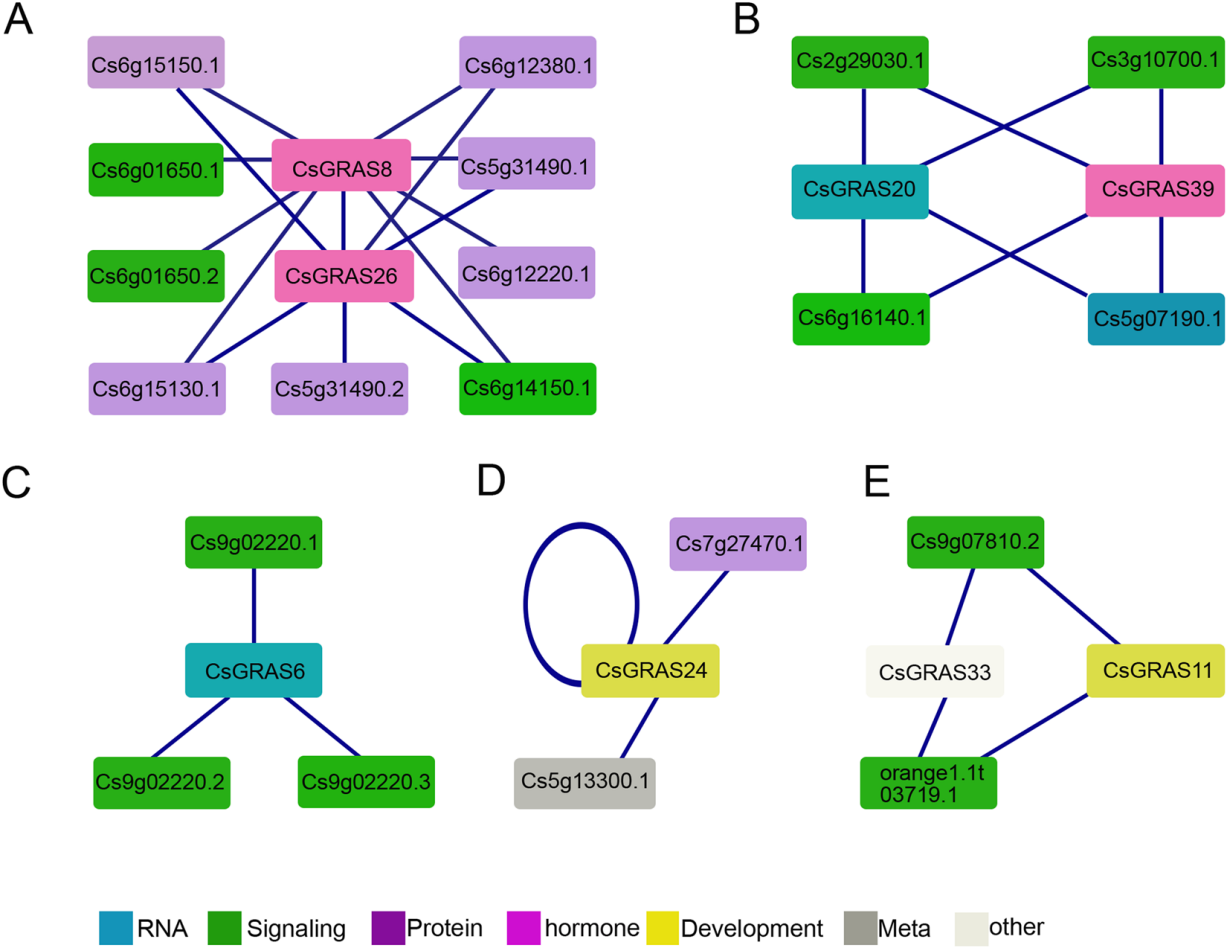
The protein-protein interactions (PPI) of GRAS protein in sweet orange. Orthologous-based and domain-based methods were employed to predict PPI network in sweet orange. The colors represent different functions of protein in rectangle. (**A**) The interacted proteins with CsGRAS8 and CsGRAS26. (**B**) The interacted proteins with CsGRAS20 and CsGRAS39. (**C**) The interacted proteins with CsGRAS6. (**D**) The interacted proteins with CsGRAS24. (**E**) The interacted proteins with CsGRAS11 and CsGRAS33.

In order to identify the function of *CsGRAS* genes, the PPI (protein-protein interact) networks of CsGRAS protein were built by *C. sinensis* annotation project database of Huazhong Agricultural University. The results showed that the PPI networks can been divided to 5 groups (Fig. 6). CsGRAS8 and CsGRAS26 were predicted to interact with each other (Fig. 6A), both of which were DELLA proteins (Fig. 2). Excluding Cs6g01650.1, other predicted interaction proteins with CsGRAS8 and CsGRAS26 belonged to F-box protein families which determine substrate specificity in the ubiquitin-proteasome pathway (Table S2). This indicates that CsGRAS8 and CsGRAS26 may be involved in the regulation of protein degradation. The DLT subfamily member CsGRAS20 and the DELLA protein CsGRAS39 had interactions with Cs2g29030.1, Cs6g16140.1, Cs3g10700.1 and Cs5g07160.1, all of which were calmodulins (Table S2). CsGRAS6 belonged to the AtPAT1 subfamily (Fig. 2), which is associated with phyA signaling^36^. Cs9g02220.1, a phyB1, and its other two transcripts interacted with CsGRAS6, indicating CsGRAS6 may function in phyB signaling. The AtSCR family member CsGRAS24 had interactions with Cs5g13300.1, Cs7g27470.1 and itself. Cs5g13300.1 is probably a rhamnose biosynthetic enzyme 1 (Table S2), and Cs7g27470.1 is elongation factor 1-alpha 1, indicating CsGRAS24 may be involved in metabolism. CsGRAS11 belonged to the AtSCL3 subfamily which functions in stress response and the GA response pathway^21^. CsGRAS33 belonged to the CsGRAS34 subfamily, of which little is known. Both interact with two calmodulin, indicating that CsGRAS33 may function in signaling via calmodulin. In short, CsGRAS family proteins may be involved in similar pathways with their homologous genes, but might also play different roles from homologous genes in Arabidopsis and rice.

The expression data of 8 GRAS proteins and 20 proposed interacted proteins were downloaded from RNA-seq library in website http://citrus.hzau.edu.cn/orange/ (Fig. S2). The expression patterns of GRAS8 and GRAS26 were consistent, the same as which of Cs6g15130.1 and Cs6g15150.1, Cs6g01650.1 and Cs6g01650.2, Cs5g31490.1 and Cs5g31490.2 (Fig. S2A). The expression patterns of GRAS20 and Cs3g10700.1 were similar in tissues of callus, flower and leaf (Fig. S2B). The genes coded GRAS6 and Cs9g02220.1 were expressed almost similarly in all the tissues checked (Fig. S2C). But the gene expression of GRAS24 is much different with which of two proposed interacted proteins (Fig. S2D). The genes coded GRAS11 and its proposed interacted proteins Cs9g07810.2 and orange1.1t03719.1 expressed much higher in callus than the other three tissues (Fig. S2E). The similar expression patterns suggested the interaction between these GRAS proteins and proposed interacted proteins was possible. However, the predicted protein interaction needs further experiments like as yeast two hybrids, pull down or Co-IP for testing.

### Cis-element analysis in the promoters of *CsGRAS* genes

In response to phytohormones and stress factors, cis-elements in promoter regions affect gene expression to regulate plant development and adaption to environmental changes. In order to obtain more information about functions of *CsGRAS* genes in citrus, 50 promoters of *CsGRAS* genes were analyzed using the online software PlantCARE.

Various types of cis elements were identified in the promoter regions, including stress responses elements related to heat and drought, and hormone response elements related to ethylene and ABA signaling (Table S3). In addition, each promoter of *CsGRAS* genes contains more than one response element. 35 promotors of 50 *CsGRAS* genes include an anaerobic stress cis-element, many *CsGRAS* promoters contain cis-elements for drought stress, SA, JA, and ABA, and numerous promoters also contain heat, fungi, ethylene, GA and IAA cis-elements. In addition, several promoters have the low temperature, anoxic and wound cis-elements. This suggests that *CsGRAS* genes may play important roles in many developmental processes and are involved in various stress responses.

### Expression profiles of *CsGRAS* genes in response to phosphorus deficiency in the root of Pt

In agricultural production, sweet orange is generally used as a scion. Pt is the main rootstock for sweet orange because of favorable growth characteristics, such as strong growth, strong root system, high survival rate, cold resistance, drought resistance and resistance to pests and diseases. Pi is an essential macronutrient for plant growth and development, and the *CsGRAS* gene family is involved in responding to Pi deficiency. For example, *AtSHR* and *AtSCR* genes have been functionally characterized in Arabidopsis under Pi deficiency^59^. It was reported that the transcriptional level of multiple transporters and some transcription factors, such as GRAS, NAP, CCAAT-binding and GATA TFs, changed under P-deficiency in Pt^33^.

**Figure 7:**
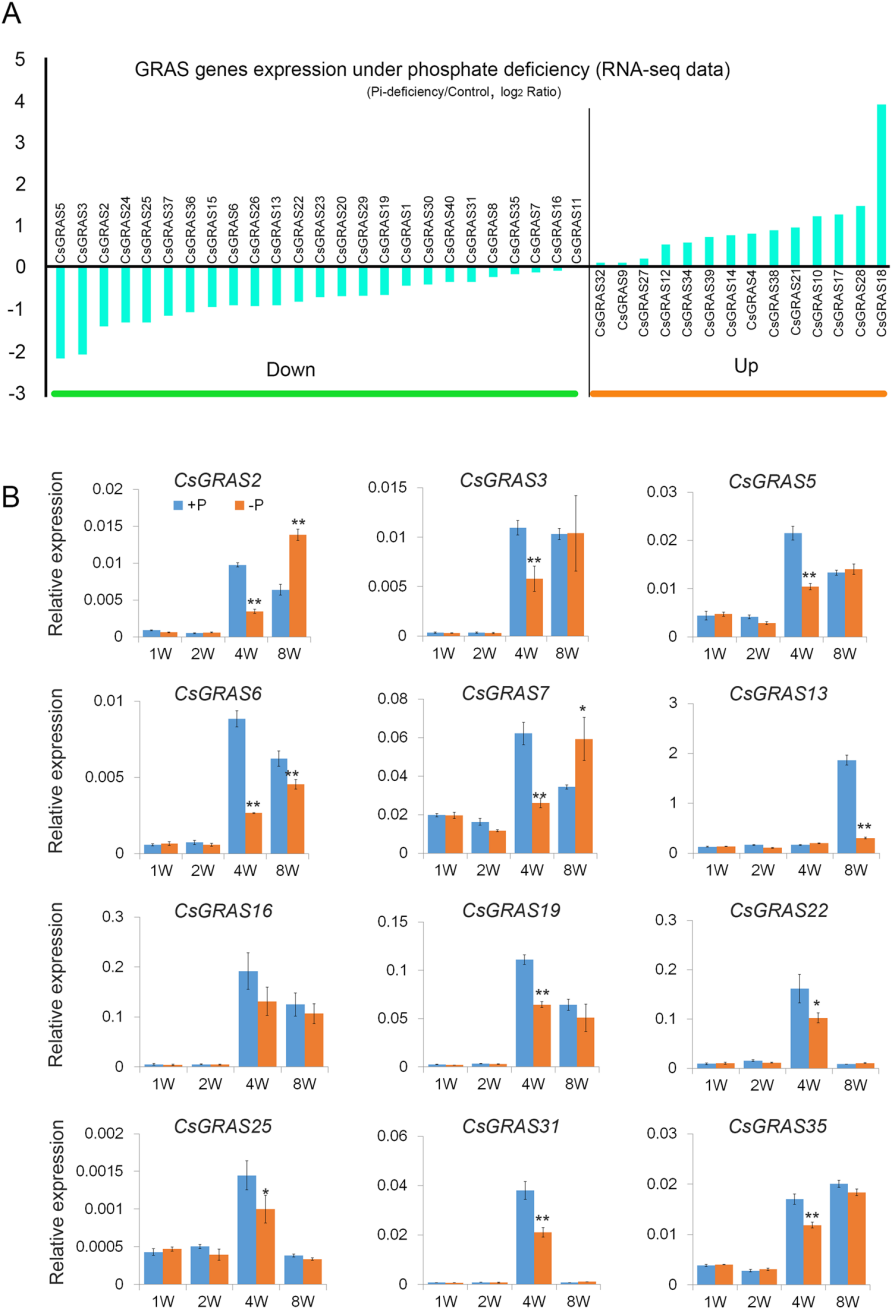
The *GRAS* genes expression under Pi-deficiency in *P. trifoliata*. (**A**) The value was RNA-seq (RPKM) which deal with the Pi-deficiency data divided by the control data, and was transformed by log2 fold change. (**B**) Expression level of 12 *CsGRAS* genes under Pi-deficiency treatment at 1W, 2W, 4W and 8W, with the Citrus actin gene as internal control Error bars denotes the standard deviation calculated from three independent experiments, statistical significance were analyzed by Student’s t-test (∗∗p < 0.01, *p < 0.05).

In this study, we investigate the expression patterns of *CsGRAS* genes in response to Pi deficiency by RNA-seq data analysis in *P. trifoliata* and quantitative real-time PCR. Firstly, expression data for all 39 *CsGRAS* genes under Pi deficiency treatment for 4 weeks in the root of *P. trifoliata* were downloaded from the report^33^. Log2-transformed expression values were used to create the histogram (Fig. 7A), and expression changes of more than two-fold were considered significant under Pi deficiency treatment. The *CsGRAS* genes were divided into two groups according to changes in expression levels under Pi deficiency: the ‘up-regulated’ group consisted of 14 *CsGRAS* genes that were upregulated by more than one-fold (on a log2 scale); 25 *CsGRAS* genes made up the ‘down-regulated’ group containing genes that were downregulated by more than one-fold (on a log2 scale) (Fig. 7A). The results revealed that 7 *CsGRAS* genes (*CsGRAS5, CsGRAS3, CsGRAS2, CsGRAS24, CsGRAS25, CsGRAS37* and *CsGRAS36*) showed significantly decreased abundance, especially *CsGRAS5* and *CsGRAS3* which were reduced more than four-fold, while 4 *CsGRAS* genes (*CsGRAS10, CsGRAS17, CsGRAS28* and *CsGRAS18*) were notably up regulated under Pi deficiency. In particular, the highest up-regulated gene was *CsGRAS18*, which increased more than six-fold. Intriguingly, the significantly up regulated genes *CsGRAS10*, *CsGRAS18* and *CsGRAS28*, all belonged to the DELLA subfamily.

It was found that Pi deficiency promoted the accumulation of a DELLA protein (RGA) and caused a reduction of bioactive GA and attenuation of GA metabolism in root cells of Arabidopsis^60^. In our study, four *CsGRAS* genes (*CsGRAS10, CsGRAS17, CsGRAS28* and *CsGRAS18*), were significantly up-regulated under Pi deficient conditions in RNA-seq data (Fig. 7A), and three of these genes (*CsGRAS10, CsGRAS18* and *CsGRAS28*) belonged to the DELLA subfamily. However, *CsGRAS8* and *CsGRAS26*, homologous to RGA and GAI, were down-regulated (Fig. 7A), indicating that these members of the DELLA subfamily may be involved in DELLA-GA signaling under Pi deficiency.

Also, *CsGRAS3* and *CsGRAS2*, *CsGRAS24* and *CsGRAS25* were part of AtSCR subfamily respectively (Fig. 2). In Arabidopsis, AtSCR and AtSHR proteins play a key role in root radial patterning^52^. PDR2 (PHOSPHATE DEFICIENCY RESPONSE 2), encoding the P5-type ATPase, was reported to restrict AtSHR movement and maintain AtSCR level during Pi deprivation^59^. *CsGRAS2*, which is homologous to AtSHR, showed decreased expression levels under Pi-deficiency suggesting that *CsGRAS2* may be involved in Pi deficiency response in the root of woody plants (Fig. 7A).

These results appeared consistent with earlier studies in Arabidopsis and crops, which demonstrated that Pi deficiency affected root development by reducing growth of primary roots while increasing the number and length of lateral roots and root hairs^34^.

We then examined the transcriptional level of 35 *CsGRAS* genes under Pi deficient conditions lasting one week, two weeks, four weeks and eight weeks (Figs 7B and S3). We found that The *CsGRAS* genes revealed time-dependent differences in expression (Figs 7B and S3), demonstrating that plants response to Pi deficiency is a complex process. The *CsGRAS* genes participating in regulating plant response to phosphorous deficiency, functioned in multiple pathways. Most *CsGRAS* genes showed lower expression level with Pi starvation treatment in the first two weeks than that at the fourth and eighth week, especially at the fourth week, such as *CsGRAS9, CsGRAS14*, *CsGRAS22* and *CsGRAS31* (Figs 7B and S3). In addition, the expression level of *CsGRAS2*, *CsGRAS3*, *CsGRAS5*, *CsGRAS6*, *CsGRAS7*, *CsGRAS*13, *CsGRAS16*, *CsGRAS19*, *CsGRAS22*, *CsGRAS25*, *CsGRAS31* and *CsGRAS35* decreased with Pi starvation treatment at the fourth week (Fig. 7B), which was consistent with the RNA-seq data analysis (Fig. 7A). Moreover, *CsGRAS2*, *CsGRAS27*, *CsGRAS32* and *CsGRAS33* showed reduced transcriptional abundance at fourth week under Pi deficiency, while an increased transcriptional abundance at the eighth week (Figs 7B and S3). Clear increasement was detected of *CsGRAS23* at the fourth week under Pi starvation treatment, which was inconsistent with the RNA-seq data (Figs 7A and S3). And some genes showed obvious response until eighth-week Pi starvation treatment, such as *CsGRAS4*, *CsGRAS13* and *CsGRAS15* (S3). In conclusion, the expression of *CsGRAS* genes exhibited a time-dependent response to Pi deficiency in trifoliate roots.

### Expression profiles of *CsGRAS* genes in response to GA_3_ and NaCl treatment

**Figure 8:**
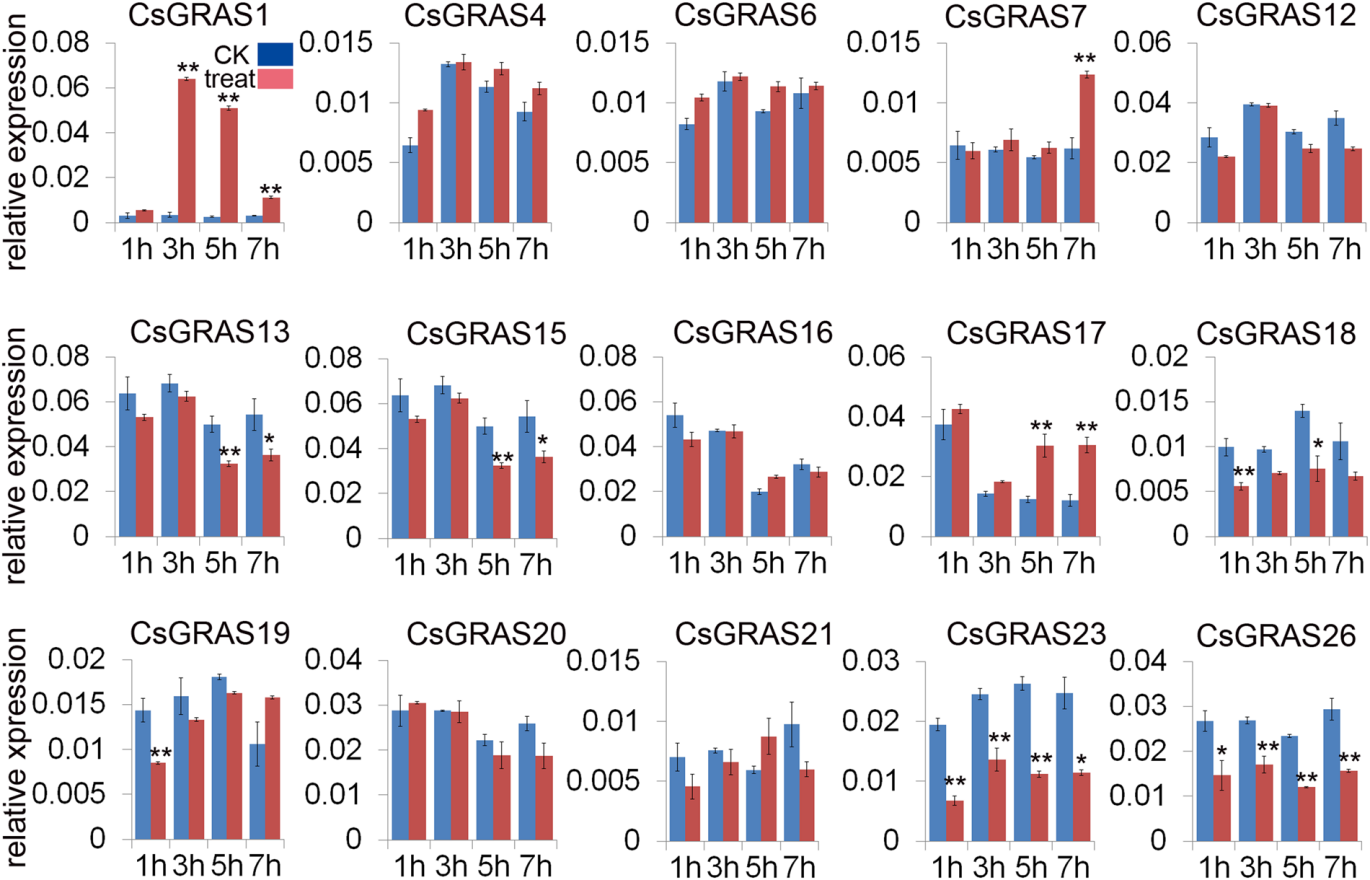
Differential expression detected for 15 *CsGRAS* genes response to GA treatment at 1 h, 3 h, 5 h, and 7 h (marked treat in red), with water treatment as control (marked CK in blue). The relative expression of selected GRAS genes under 100 mM NaCl was determined by qRT-PCR. The Citrus actin gene was used as internal control. Error bars denotes the standard deviation calculated from three independent experiments, statistical significance were analyzed by Student’s t-test (**p < 0.01, *p < 0.05).

GA_3_ plays a critical role in plant growth and development, and GRAS proteins in Arabidopsis and rice participate in GA responses^16–19^. To investigate *CsGRAS* gene expression in response to GA treatment, qRT-PCR was performed to analyze changes in the expression levels of 15 *CsGRAS* genes in GA treatment at 1 h, 3 h, 5 h and 7 h, with water treatment as control. Three genes (*CsGRAS1*, *CsGRAS 7* and *CsGRAS* 17) were up-regulated at 3 h, 7 h and 5 h during GA treatment, respectively, whereas *CsGRAS18*, *CsGRAS*19, *CsGRAS23* and *CsGRAS26* were all down-regulated at 1 h, *CsGRAS13* and *CsGRAS15* showed lower expression level at 5 h and 7 h under GA treatment (Fig. 8). Moreover the promoters of *CsGRAS1, CsGRAS13*, *CsGRAS15, CsGRAS23* and *CsGRAS26* have the response element to gibberellin (Table S3), indicating that certain *CsGRAS* genes could be involved in the GA signaling pathway.

**Figure 9:**
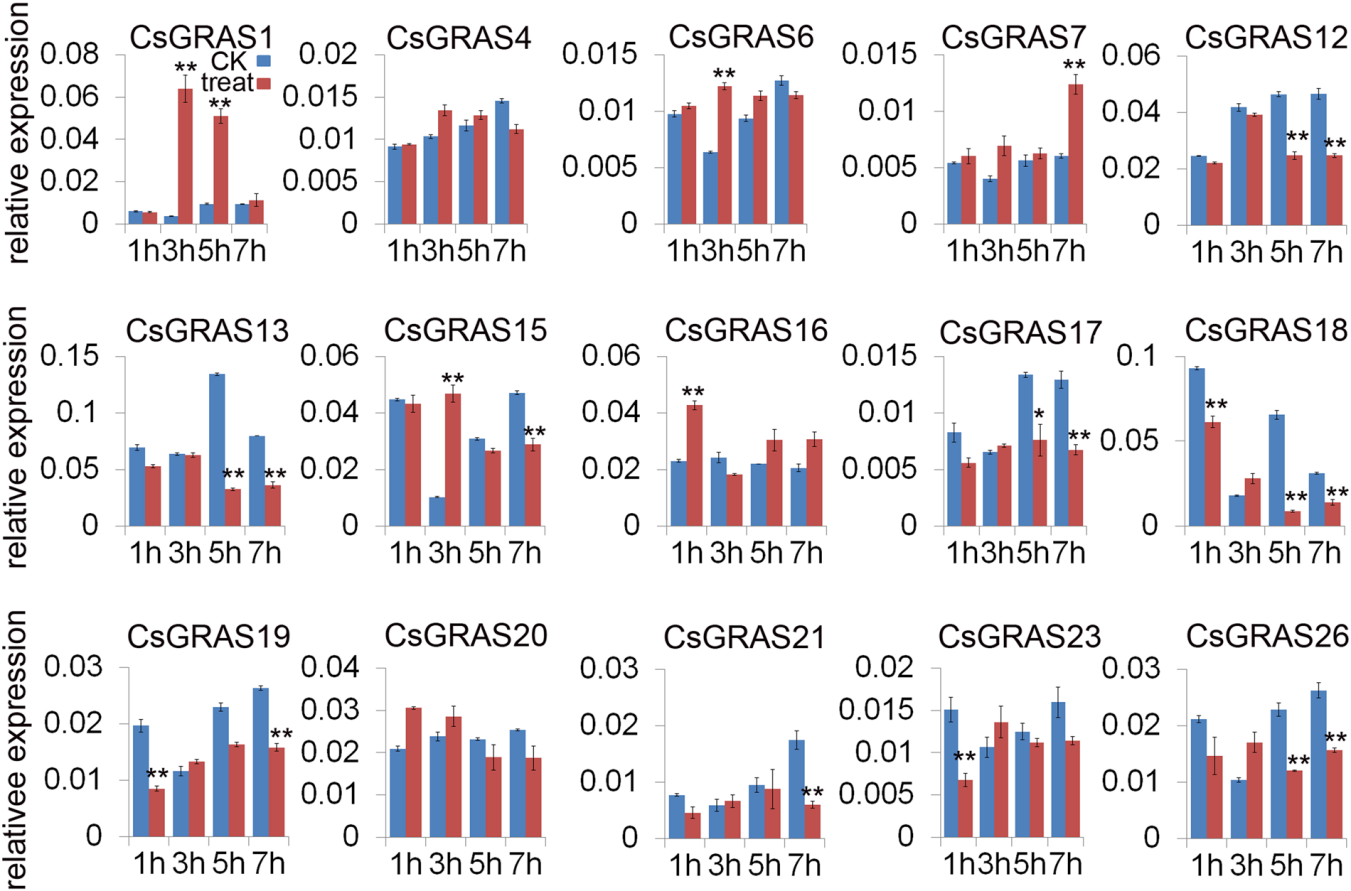
Differential expression detected for 15 *CsGRAS* genes response to NaCl treatment at 1 h, 3 h, 5 h, and 7 h (marked treat in red), with water treatment as control (marked CK in blue). The relative expression of selected GRAS genes under 100 mg/L GA3 was determined by qRT-PCR. The Citrus actin gene was used as internal control. Error bars denotes the standard deviation calculated from three independent experiments, statistical significance were analyzed by Student’s t-test (**p < 0.01, *p < 0.05).

Previous studies showed that GRAS proteins function in abiotic stress^40,41^, therefore we checked the transcriptional level of *CsGRAS* genes in response to NaCl treatment. *CsGRAS1* showed increased expression level at 3 h, and *CsGRAS7* at 7 h, similar to their response to GA treatment (Fig. 9). *CsGRAS6* and *CsGRAS1*5 were up-regulated at 3 h, while *CsGRAS16* was upregulated at 1 h (Fig. 9). Interestingly, *CsGRAS18*, *CsGRAS23* and *CsGRAS26* were down-regulated in response to NaCl treatment, similar to their expression changes under GA treatment (Fig. 9). *CsGRAS* genes (*CsGRAS 12*, *13*, *17*, *19* and 21) also showed lower transcriptional level than control at different NaCl treatment times, in comparison *CsGRAS17* showed higher expression levels under GA treatment (Fig. 9). Collectedly, more *CsGRAS* genes were affected by NaCl treatment than by GA treatment. Some genes may play roles in both NaCl and GA responses, while some genes may show opposite roles under different treatments.

Many GRAS members have been found to be involved in GA signal regulation in Arabidopsis, rice and other species. RGA, GAI, RGL1, RGL2 and RGL3 are negative regulators of the GA signal in Arabidopsis^27^. Rice GRAS protein, SLR1, is known to be involved in GA signaling^55^, and CIGR1 and CIGR2, belonging to the rice GRAS family, are rapidly induced by exogenous gibberellins^39^. Moreover, SlGRAS24, target of tomato miR171, showed high sequence identity with AtSCL6^47^, which is involved in GA and auxin signaling^61^. CsGRAS18 and CsGRAS26, which are members of DELLA subfamily, showed lower transcriptional level than control at 1 h, 3 h, 5 h and 7 h under GA3 treatment (Fig. 8). In addition, CsGRAS7 and CsGRAS17, which are in the same subfamily as OsCIGR1 and OsCIGR2, could be induced by GA3 at 7 h and 5 h respectively (Fig. 8). Therefore, the CsGRAS proteins may play similar roles as other proteins in the same subfamily. All the genes affected by GA_3_ were up-regulated or down-regulated under 100 mM NaCl treatment, implying that these genes could be involved in both GA signaling and stress response.

### Conservation of GRAS subfamily in different species

To investigate the distribution and conservation of the *GRAS* gene subfamilies, we statistically analyzed gene identities among six species. Using Arabidopsis as reference, we chose 9 *GRAS* genes in Arabidopsis and two genes from rice and *C. sinensis* as a query to blast the most homologous genes in the other 5 species.

From peptide analysis of 6 species (*Populus trichocarpa, Oryza sativa, Physcometrella patens, Selaginella moellendorffii, A. thaliana*, and *C. sinensis*) (Fig. S4), *AtPAT1* and *AtSCR* seemed the most conserved of the GRAS subfamilies, related to fundamental functions, such as phytochrome signaling, root radial patterning and maintenance of QC identity. We discovered genes orthologous to *AtPAT1* among the 5 species and orthologous to *AtSCR* among 4 species (Fig. S4). The proteins of these two subfamilies shared high identity and contained extremely conserved fragments, indicating that they originated from one ancestor, with minimal change through evolution. AtSCL3, DELLA, DLT, AtSHR and LISCL subfamilies were highly conserved among the six species, especially among the dicotyledonous *Arabidopsis, Citrus* and *Populus* (Fig. S4). AtLAS & At4/7 were highly conserved among *Arabidopsis, Citrus* and *Populus*, and moderately conserved among the other three species (Fig. S4). However, there was no AtHAM subfamily in *P. patens, S. moellendorffii*, and *O. sativa*, but which conserved in *C. sinensis* and *Populus*. Os19 subfamily was discovered in *C. sinensis, O. sativa* and *Populus*, and was absent in the other three species. The CsGRAS34 subfamily of *C. sinensis* also existed in *Populus*, but was absent in the other four species. This indicates the CsGRAS34 subfamily may exist specifically in woody plants.

Comparison of the subfamilies from 6 species revealed subfamily conservation between species (Fig. S4). Certain subfamilies were species specific. Os19 only existed in rice, *C. sinensis* and Populus. While CsGRAS34 existed only in *C. sinensis* and populus, indicating that these subfamilies originated simultaneously with species differentiation in evolution. On the other hand, the subfamily conservation analysis confirmed high homology between citrus and populus.

## Conclusion

In conclusion, a total of 50 *CsGRAS* genes were identified and characterized in the sweet orange genome, and were phylogenetically classified into 11 distinct subfamilies. We found segmental duplications and tandem duplication had contributed to the expansion of CsGRAS family. Moreover, some *CsGRAS* genes appeared to be differentially expressed in different tissues, and expression levels of some *CsGRAS* genes were influenced by phosphorus deficiency, salt stress, and GA_3_ treatment. These data will provide the basis for understanding evolutionary history and the developmental roles of CsGRAS proteins in sweet orange, and may be helpful for future exploration of the biological functions of *CsGRAS* genes. These findings will also serve as a resource for identifying genes that improve citrus growth under stress conditions and enable potential breeding and genetic improvements for agriculture.

## Materials and Methods

### Identification and Chromosomal Locations of *GRAS* genes in *C. sinensis*

To identify all *GRAS* genes in orange (*C. sinensis*), all annotated proteins were downloaded from *C. sinensis* annotation project database of Huazhong Agricultural University (http://citrus.hzau.edu.cn/orange/) and the phytozome *Citrus sinensis* v1.1 database (http://phytozome.jgi.doe.gov/pz/portal.html). The Hidden Markov Model (HMM) profile of the GRAS domain (PF03514) downloaded from Protein family (Pfam) (http://pfam.sanger.ac.uk/) was used for identification of the *GRAS* genes from the downloaded database of orange genome using HMMER3.0. All output genes with default (<1.0) E-value were collected and the online software SMART (http://smart.embl-heidelberg/) was used to confirm the integrity of the GRAS domain with E-value < 0.1, and the incorrectly predicted genes were rejected. Finally, the non-redundant and confident genes were gathered and assigned as orange *GRAS* genes. The physical positions of *CsGRAS* genes were obtained from *Citrus sinensis* annotation project database of Huazhong Agricultural University and the phytozome *Citrus sinensis* v1.1 *Citrus sinensis* database. Motif location of GRAS protein in *C. sinensis* was discovered by online program MEME (http://meme-suite.org/tools/meme).

### Multiple sequence alignment and phylogenetic analysis of *GRAS* genes in *C. sinensis* combined with rice and Arabidopsis

Multiple sequence alignment was executed by ClustalX 2.0 program and GeneDoc. Phylogenetic trees were constructed using MEGA 6.0 by the Neighbor-Joining (NJ) methods and the bootstrap test carried out with 1000 iterations.

### Putative cis-elements in the promoter regions

The 1500 bp upstream sequences from the translation start codon of all of the *CsGRAS* genes were obtained from *Citrus sinensis* annotation project database of Huazhong Agricultural University (http://citrus.hzau.edu.cn/orange/). The putative stress or hormone responsive cis-acting regulatory elements in these sequences were predicted using the PlantCARE web server http://bioinformatics.psb.ugent.be/webtools/plantcare/html/)^62,63^ and then to identify the putative cis-acting regulatory elements.

### Expression profiles of *GRAS* gene family in *C. sinensis* by RNA-seq analysis

Expression profile data of *CsGRAS* gene family in 4 tissues for sweet orange were extracted from *C. sinensis* annotation project database of Huazhong Agricultural University (http://citrus.hzau.edu.cn/orange/)^1^. Three independent samples and libraries sequenced for each of the tissues were used. The data was calculated by reads per kilobase per million mapped reads (RPKM) as transcript abundance and the RPKM values were transformed in log10 fold change. The heat map generation and cluster analyses were performed using R v3.3.0. To validate the accuracy of the RNA-seq data, three independent samples for each tissue (leaf, callus, flower, and fruit) were collected, then the RNA was extracted and the expression levels of some selective *CsGRAS* genes in sweet orange tissues by qRT-PCR were investigated.

### RNA extraction and real-time quantitative PCR analysis

Total RNA was extracted using TransZol Reagent (TransGen Biotech, China) according to manufacturer instructions. RNA integrity was verified by 1% agar gel electrophoresis and the RNA concentration was measured using NanoDrop (2000, USA). First-stand cDNA was synthesized from 4 μg total RNA using the TransScript® One-step gDNA Removal and cDNA Synthesis SuperMix Kit (TransGen Biotech, China) following manufacturer protocols. Real-Time quantitative PCR was carried out on an Applied Biosystems®QuantStudioTM 7 Flex Real-Time PCR System (life technologies, USA) using 2 × HSYBR qPCR Mix (With ROX II) (ZOMANBIO, China). Each reaction was performed in a 10 μl volume containing 5 μl 2 × HSYBR qPCR Mix, 0.4 μl template DNA (1–10 ng cDNA), 0.2 μl each primer (10 μM), and finally adding the RNase-free water to give a total volume of 10 μl. The PCR amplification cycle was as follows: 95 °C for 10 min, 40 cycles at 95 °C for 15 s, and 60 °C for 1 min. Melting curve analysis was executed for verifying the specificity of primer with the following program: 95 °C for 15 s, 60 °C for 1 min, 95 °C for 15 s, 60 °C for 15 s. All quantitative Real-Time PCR experiments were conducted in three biological replicates. Relative fold differences were calculated based on the comparative Ct method using the 2−△△Ct method with ACTIN2 as the internal reference gene. All the primers (Table S4) for qRT-PCR were designed based on the reference sequence obtained from the *C. sinensis* Annotation Project (http://citrus.hzau.edu.cn/orange/).

### PPI network of GRAS protein in *C. sinensis*

PPI (protein-protein interact) network of GRAS protein in *C. sinensis* was built. Orthologous-based and domain-based methods were employed to predict PPI network in *C. sinensis* annotation project database of Huazhong Agricultural University (http://citrus.hzau.edu.cn/orange/), which was shown by Cytoscape 3.3.0.

### Plant Growth and Treatment with phosphorus deficiency in *P. triforniata*

A common rootstock, *P. triforniata* (L) Raf (Pt), was used in this study. Pt seeds were sown in plastic pots filled with vermiculite as previously described. Twenty days later, uniform seedlings were transplanted into sand culture. Pt seedlings in sand pots were grown in a chamber with a 14 h light period at 23–28 °C and a 10 h dark period at 18–20 °C. Five Pt seedlings per sand pot were irrigated with 200 ml Hoagland nutrient solution. Two-month-old seedlings were used for Pi starvation treatment. The control samples (+P) were irrigated with Hoagland nutrient solutions containing 1 mM P, whereas P starvation samples (−P) were irrigated with Hoagland nutrient solutions containing 1 μM P^33^. Root samples were collected at 0, 1, 2, 4, 8, or 10 weeks after (−P) irrigation treatment. Each root sample was prepared from three pots with each containing five seedlings. Three root samples/per treatment were collected for each time point from three independent experiments^33^.

### Expression of *GRAS* genes in Pt under phosphorus deficiency via analysis RNA-seq profiles

We selected expression profile data of *GRAS* genes from the transcriptome sequencing using Illumina HiSeqTM2000 under Pi deficiency treatment for 4 weeks^33^. The data was calculated by reads per kilobase per million mapped reads (RPKM) as transcript abundance and the RPKM values were transformed in log2 fold change values in Microsoft excel 2013. To validate the accuracy of the RNA-seq data, we collected roots of Pt under 0, 1, 2, 4, 8, or 10 weeks after (-P) irrigation treatment and examined the expression of selected *CsGRAS* genes, which exhibited up- or down-regulation by more than two-fold, based on qRT-PCR.

### Plant growth and treatments with GA3 and NaCl in sweet orange

One-month-old sweet orange plants, grown in greenhouse at 28 °C, with 16-h light/8-h dark photoperiod, were used to examine the *CsGRAS* gene expression level under treatments. Uniform and healthy plants were selected from the plants and inserted in flasks containing 100 mg/L GA3 or 100 mM NaCl respectively, with distilled water as control. Three independent samples were collected at 1, 3, 5, and 7 h after treatment, then frozen immediately in liquid nitrogen and stored at −80 °C until using for RNA extraction.

## Supplementary information

Supplementary information

Dataset 1

Dataset 3

Dataset 2

Dataset 4

## Data Availability

All the data reported in our manuscript is available and can be found in the article and supplemental materials.
