# Supplementary material for: Genome-wide identification, characterization, interaction network and expression profile of GRAS gene family in sweet orange (Citrus sinensis)
Source: Sci Rep. 2019 Feb 15;9:2156. doi: 10.1038/s41598-018-38185-z (PMC6377710; doi:10.1038/s41598-018-38185-z)

**Genome-wide identification, characterization, interaction network and expression profile of *GRAS* gene family in sweet orange (*Citrus sinensis*)**

**Running title: Characterization of *GRAS* gene family in sweet orange (*Citrus sinensis*)**

Hua Zhang^1^, Limin Mi^1^, Long Xu^1^, Changxiu Yu^1^, Chen Li^2^, Chunli Chen^1, 3*^

^1^ College of Life Science and Technology, Huazhong Agricultural University, Wuhan, 430070, China

^2^ School of Basic Medicine, Hubei University of Medicine, Shiyan, Hubei, 442000, China

^3^ Key Laboratory of Horticultural Plant Biology (Ministry of Education), Huazhong Agricultural

University, Wuhan, 430070, China

**Supplementary Table S1. Basic information of CsGRAS family**

**Supplementary Table S2. *CsGRAS* genes function annaotation**

**Supplementary Table S3. Fig.7 Cis-elements in promoters of *CsGRAS* genes.**

**Supplementary Table S4. List of primer sequences used in qRT-PCR**

**Supplementary Figure S1. Relative expression of 35 *CsGRAS* genes in 4 tissues of sweet orange including callus, flower, leaf and fruit.** Error bars denotes the standard deviation calculated from three independent experiments.

**
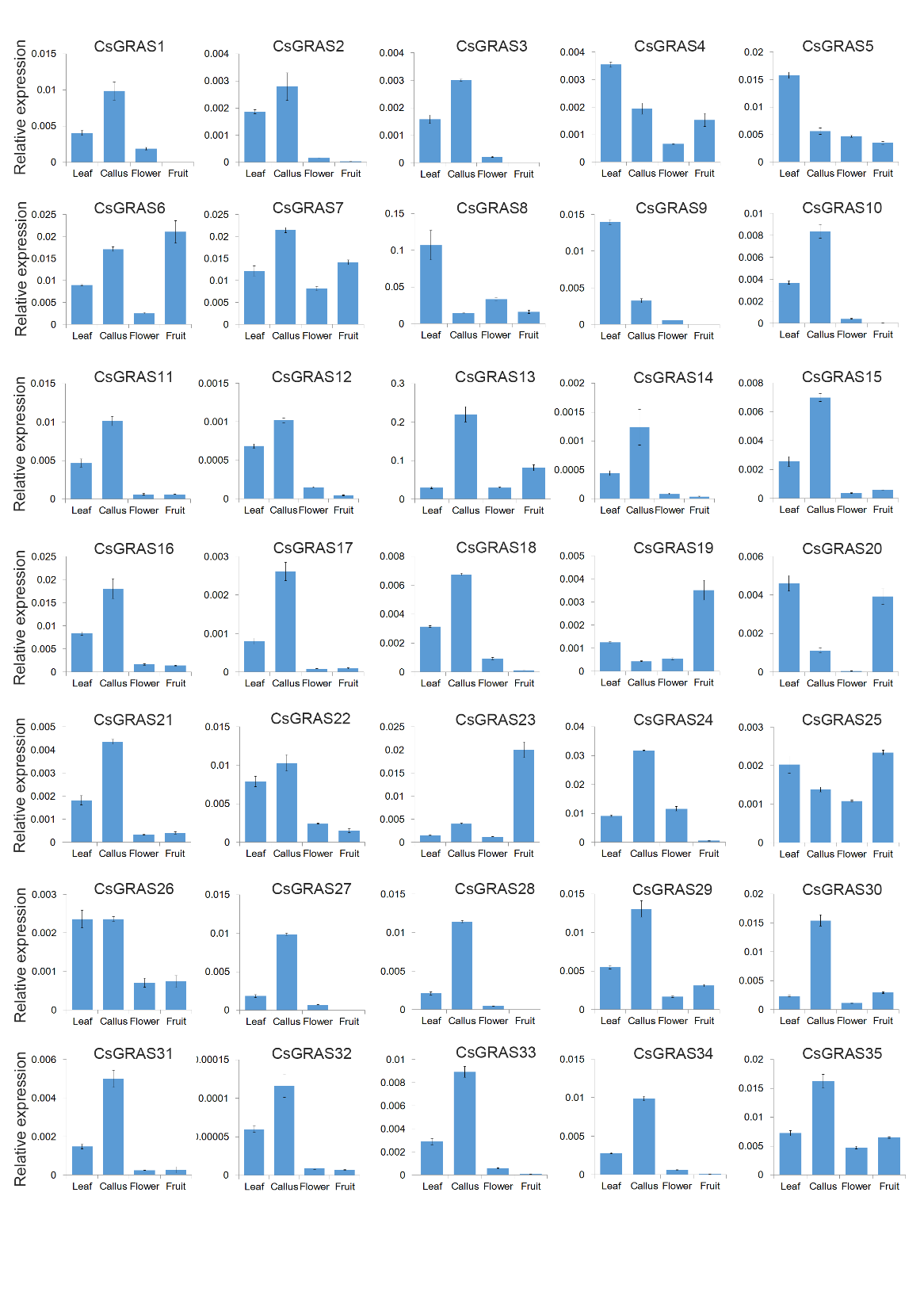
**

**Supplementary Figure S2. The genes’ expression pattern of 8 GRAS and 20 proposed interacted proteins**

**
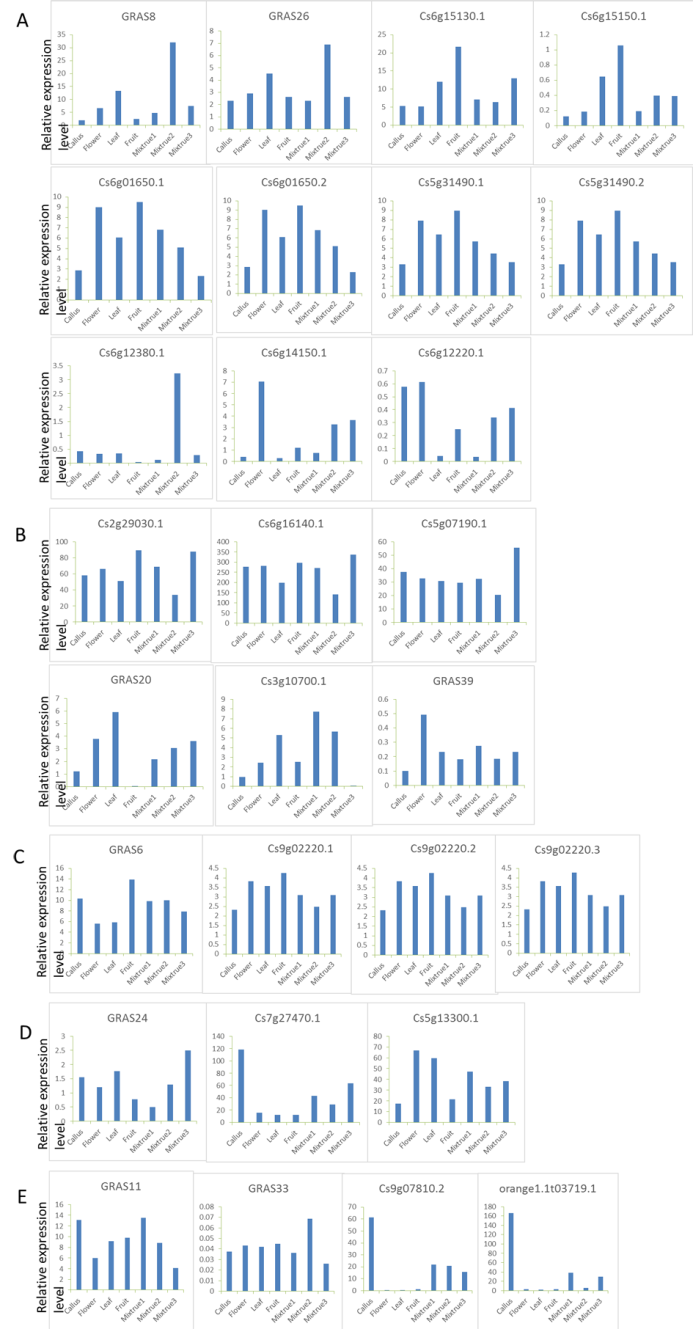
**

**Supplementary Figure S3. Expression level of 35 *CsGRAS* genes under Pi-deficiency treatment at 1 W, 2 W, 4 W and 8 W with the Citrus actin gene as internal control.** Error bars denotes the standard deviation calculated from three independent experiments, statistical significance were analyzed by Student's t-test (∗∗ p < 0.01, * p < 0.05 )

**
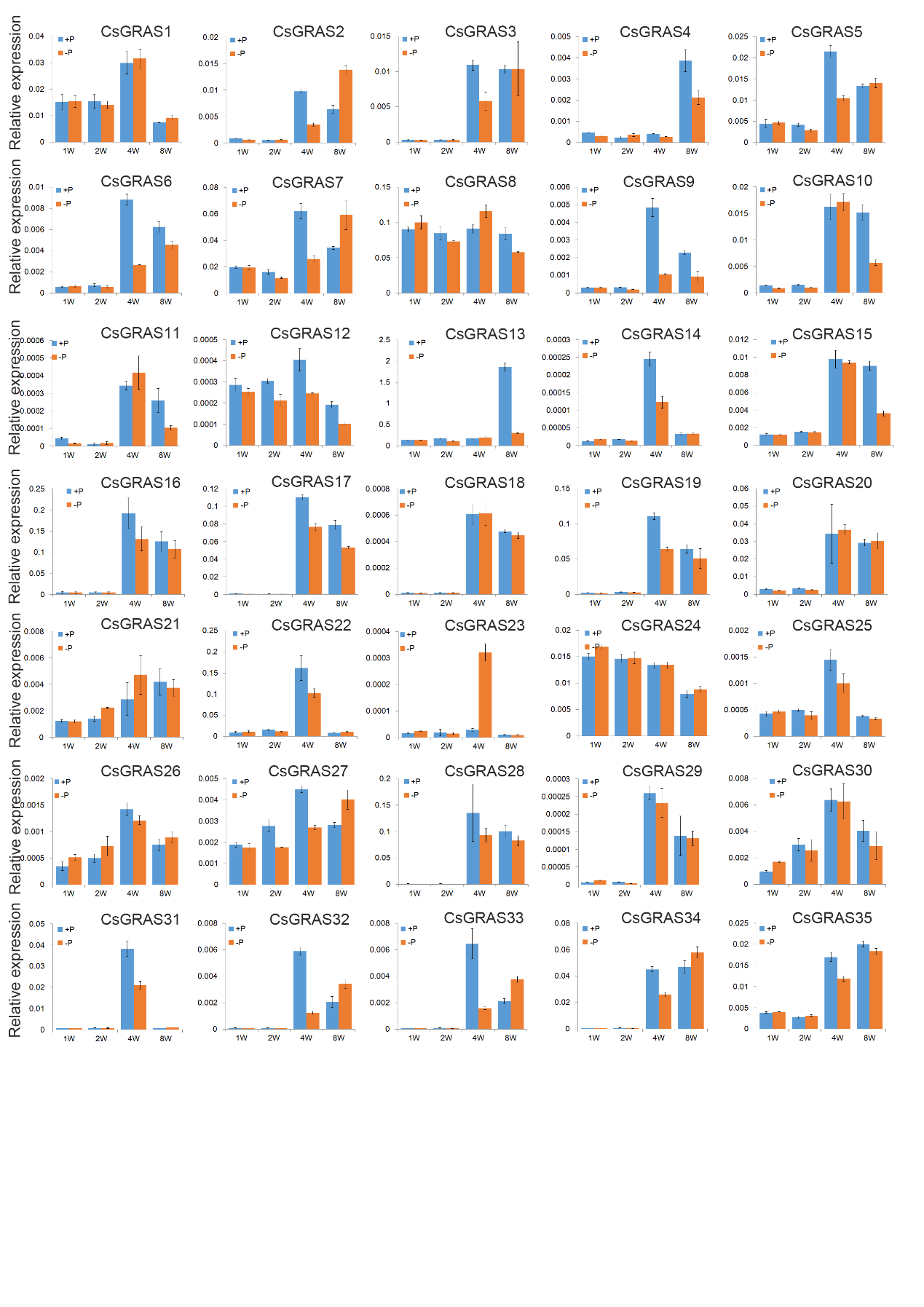
**

**Supplementary Figure S4. Conservative of GRAS subfamily in 6 species.** The white box with GRAS proteins in black color are representative of each subfamily. Colors in boxes represent the identities of each species compare to the representative GRAS proteins by blast.


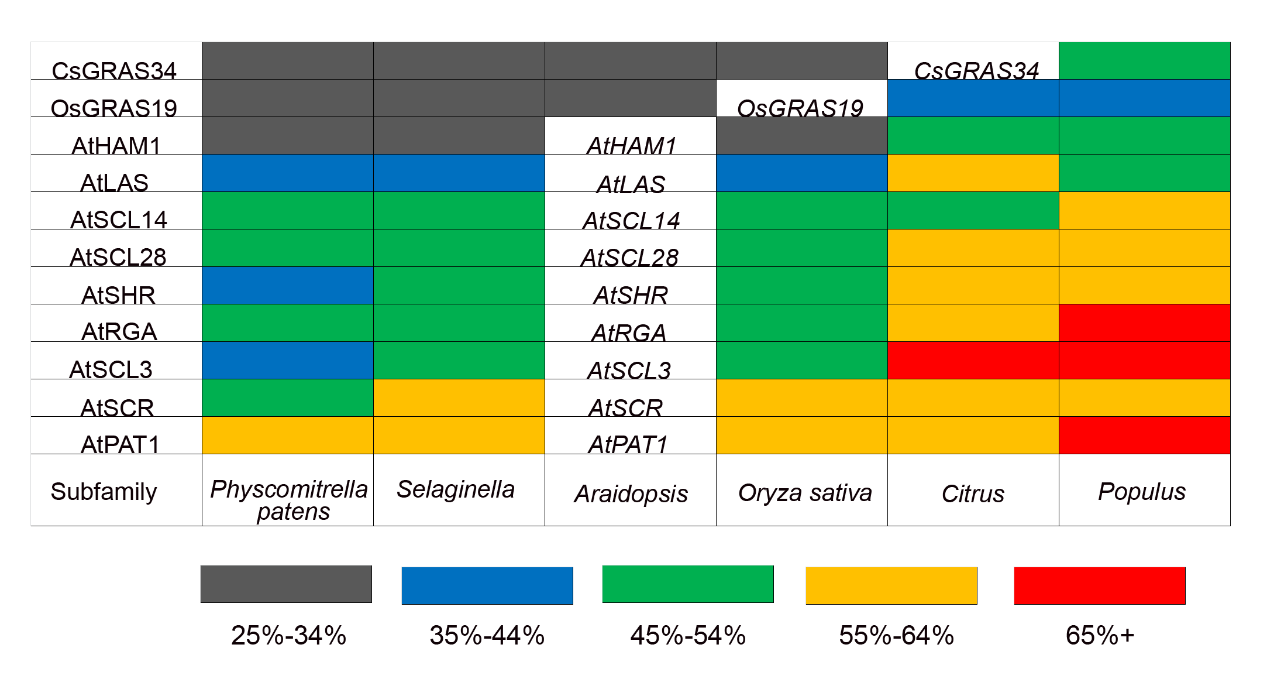

Supplement: Supplementary file 1 — Supplementary information [file 41598_2018_38185_MOESM1_ESM.docx]
